# Silicosis: from pathogenesis to therapeutics

**DOI:** 10.3389/fphar.2025.1516200

**Published:** 2025-01-29

**Authors:** Bijun Yang, Xiaoman Liu, Cheng Peng, Xiangjing Meng, Qiang Jia

**Affiliations:** ^1^ Shandong Academy of Occupational Health and Occupational Medicine, Shandong First Medical University & Shandong Academy of Medical Sciences, Jinan, Shandong, China; ^2^ Queensland Alliance for Environmental Health Sciences, University of Queensland, Brisbane, QLD, Australia

**Keywords:** silicosis, pathogenesis, therapeutics, lung fibrosis, molecular mechanisms

## Abstract

Silicosis is an important occupational lung disease caused by exposure to respirable crystalline silica dust particles, with the clinical manifestations from asymptomatic forms to respiratory failure. The main pathological process involves parenchymal lung injury, inflammation and lung tissue fibrosis, but the exact pathogenesis remains elusive. Until now, there have been no effective treatments for silicosis due to the complexity of pathogenesis and irreversibility of pulmonary fibrosis. In this review we attempt to summarize the advances in pathogenesis and treatment of silicosis and to explore the current understanding of the molecular mechanisms involving in the initiation and development of silicosis and potential therapeutic targets.

## 1 Introduction

Silicosis, a fibrotic lung disease caused by the long-term inhalation of the dust containing silicon dioxide (SiO_2_) in occupational activities (Wang M. et al., 2022), has been a major occupational disease in many developing countries and resurged recently in some developed countries (GBD 2013 Mortality and Causes of Death Collaborators, 2015) It is reported that there are 3 million workers in Europe (Kauppinen et al., 2000) and 1.7 million workers in the United States (Li et al., 2002) exposed to silica dust. In China, occupational pneumoconiosis is the most serious occupational disease. Totally 171,291 cases of silicosis have been documented up to 2021, representing 78.58% of all pneumoconiosis cases (Liu et al., 2024). Therefore, silicosis has caused a heavy burden in developing countries and seriously threatened the quality of life (QOL) of workers.

According to the exposure period and pathological progress, silicosis is categorized as acute, accelerated and chronic types (Wang M. et al., 2022; Aloe et al., 2022). Acute silicosis mainly manifests as silico proteinosis, typically occurring within a few weeks to years following exposure to high concentrations of SiO_2_ (Krefft et al., 2020). Based on the chest imaging, chronic silicosis can be divided into simple and complicated subtypes (Quan et al., 2022). Complicated silicosis, also known as progressive macro fibrosis, is diagnosed by the presence of an International Labour Organization-classified macro mixed image on a chest radiograph or an aggregated chest high-resolution computed tomography (HRCT) image, usually accompanying by calcification and fibrosis (Quan et al., 2022; Cox et al., 2014). The onset of accelerated silicosis is within 5–10 years of exposure to dust, but the disease progresses more rapidly than chronic silicosis. In recent years, accelerated and acute silicosis has attracted more attentions, but the risk factors and molecular pathogenesis remain elusive (Adamcakova and Mokra, 2021).

Silicosis is characterized by persistent inflammation and progressive fibrosis in the lung, which may cause breath difficulty and respiratory failure, even death. Therefore, the treatment strategies using drugs are mainly to block fibrotic factors, reduce lung inflammation, inhibit the proliferation and activation of interstitial cells, and regulate the synthesis and degradation of extracellular matrix (ECM), thereby reducing the lung tissue injury and formation of pulmonary fibrosis (Pang et al., 2019; Liu et al., 2016). Current prescribed drugs for silicosis include pirfenidone, poly-2-vinylpyridine-N-oxide (PVNO), nintedanib, tetrandrine, etc. (Pang et al., 2019; Ernst et al., 2002). With the understanding of the molecular mechanisms underlying silicosis, more and more signal pathways have been identified and proposed as the potential therapeutic targets.

As a surgical treatment, bronchoalveolar lavage and whole lung lavage (WLL) are effective ways to relive the symptoms of silicosis by removing the residual silica in the alveolar cavity (Cottin et al., 2004). However, the actual therapeutic effect of this method needs to be confirmed through long-term follow-up and documentation. Although lung transplantation can be used for patients with advanced silicosis, it is hard to be widely applied due to the difficulty of donor source and high cost (George et al., 2019). In recent decades, great progress has been made in stem cell technology which may be the new and promising means for silicosis after surgical and drug treatments (Chen et al., 2018a).

The aim of this article is to review the recent progress in understanding the molecular pathogenesis of silicosis, current treatment protocols, and potential therapeutic options.

## 2 Toxicity of silica dust

Free SiO_2_ particles or respirable crystalline silica (RCS) dust particles with the diameter less than 5 μm could directly produce cytotoxicity. The degree of lung injury caused by silica dust mainly depends on the physical/chemical properties and toxicities, as well as the exposure time and intensity. Silica, also called SiO_2_, has a crystalline or non-crystalline (amorphous) structure (Lee et al., 2020). In crystalline silica, silicon atoms and oxygen atoms are arranged in a fixed geometric pattern. In contrast, there is no spatial order of atoms in amorphous silicon (Woźniak and Wiecek, 1995). It has been well documented that exposure to silica dust can cause tissue injury, inflammation, and pulmonary fibrosis. Epidemiological and laboratory studies showed that exposure to silica dust could cause lung cancer and tuberculosis (Wang D. et al., 2020). Amorphous silica is considered less toxic than crystalline silica (Rubio et al., 2019) classified as group A carcinogens by the International Agency for Research on Cancer (Guha et al., 2011; Peters et al., 2017), although its carcinogenicity to humans has not been confirmed.

In recent decades, artificial or engineered stones have been increasingly popular materials used to fabricate kitchen and bathroom bench tops. Exposure to RCS from artificial stones can increase the risk of silicosis in developed countries, such as Italy, Spain, Israel and United States (Kramer et al., 2012; Pérez-Alonso et al., 2014; Paolucci et al., 2015). Compared with traditional silicosis, artificial stone silicosis is more aggressive because of the high content of silica (90%) in the RCS (Ophir et al., 2016). Studies on the dust from artificial stones further confirmed the importance of the physical/chemical properties in their toxicities and consequent silicotic potency. It has been found that the higher contents of SiO_2_ in the dust are, the more toxic to the lung tissue is Lee et al. (2020); the higher dispersion of silica dust particles and the larger proportion of fine particles are, the greater amount of silica dust entering the deep respiratory tract is Fernández Álvarez et al. (2015).

RCS can penetrate deep into the lung with various biological activities. Silica particles with a diameter of less than 3.2 μm account for 100% of the lung tissue in silicosis patients. The silanol groups on the surface of silica dust can form hydrogen chains with secondary lysosomal membrane proteins in macrophages, which can increase the membrane permeability, reduce fluidity, and promote the membrane lysis (Goumans et al., 2008). A subfamily of silanol “nearly free silanol” is the major determinant of silica-induced toxicity through interaction with the cell membrane or phagolysosome promoting membranolysis (Pavan et al., 2020). In addition, the silica dust can directly and indirectly induce reactive oxygen species (ROS) in alveolar macrophages (AMs) to activate the cell-signaling pathways launching cytokine release and apoptosis (Hamilton et al., 2008). The human body mainly clears the dust in the respiratory tract through the ciliary oscillation of the ciliary adhesive device and the phagocytosis of AMs. However, long-term inhalation of dust will reduce the body’s defense function, which can lead to excessive deposition of dust to damage the lung tissue, thereby causing the disease (Wildung et al., 2022; Noël et al., 2020).

## 3 The pathogenesis of silicosis

**FIGURE 1:**
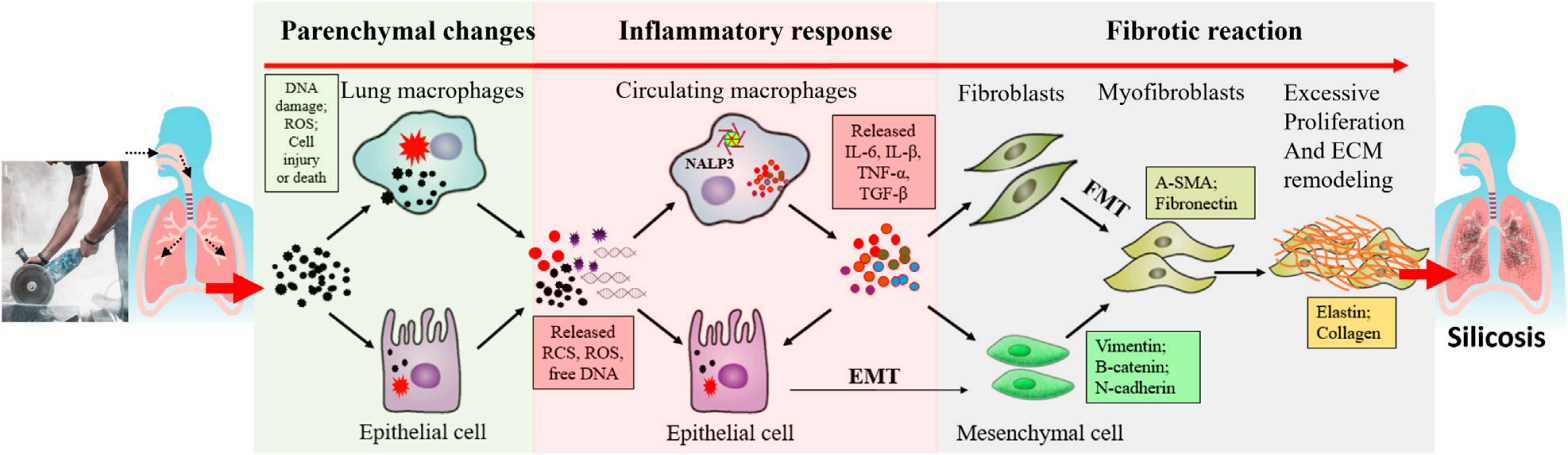
A general schematic diagram describing the different stages of silicosis. Silica dust exposure can activate macrophages, damage alveolar epithelial cells and then initiate inflammation reactions. These inflammatory factors induce multiple signaling pathways, such as TGF-β/Smad and TNF-α signaling pathways. As epithelial cells are damaged, they undergo autophagy and apoptosis. Repeated inflammation and cell death promote the formation of fibroblasts and myofibroblasts, produce lots of extracellular matrices, accelerate the release of fibroblasts and epithelial-mesenchymal transition process, thus resulting in the destruction of alveolar structures and fibrosis.

Although great efforts have been made to understand the pathogenesis of silicosis, the molecular mechanism underlying the initiation and development of the disease remains to be elucidated. The pathological process is complex and involves multiple cell types, cytokines and pathways. To simplify the complexity, we divide this process into different stages, including parenchymal lung injury, inflammatory response, and pulmonary fibrosis (Krefft et al., 2020; Kawasaki, 2015) (Figure 1). It should be noted that in real situations, these stages are not separated but overlapped and interactive.

### 3.1 Parenchymal lung injury

The human respiratory system has a strong ability to defend and remove dust through multi-level defense (de Lima Gondim et al., 2019). The initial line of defense consists of the retention function of nasal hairs and passages, which can block and trap larger particles. The second line of defense is provided by the excretory action of the mucosal ciliary system within the epithelium of the respiratory tract. The mucosal epithelium contains mucus and cilia supported by ciliated cells, which can rapidly swing toward the larynx and transport mucus and attached particles to the pharynx, where they are ultimately expelled from the body through coughing or swallowing. This mechanism effectively removes the particles with diameters ranging from 1 to 5 microns. The final line of defense is established through the phagocytosis performed by lung macrophages. These macrophages, along with other immune cells, are capable of engulfing and digesting smaller particulate matter, particularly those smaller than 2.5 microns. They are present in abundance in the alveoli and function as scavengers (Aloe et al., 2022; Su Y. et al., 2020; Fèvre et al., 2022; Nardi et al., 2018). As the resident cells in lung tissues, AMs play a major role in maintaining immune homeostasis and host defense (Cui et al., 2022), and is the critical line of defense against silica dust. AMs are derived from bone marrow mononuclear cells, with the functions of engulfing foreign bodies, anti-infection, regulating inflammatory and immune responses (Hou et al., 2019). Silica dust is recognized by AMs mainly through class A and B1 scavenger receptors, and then swallowed by AMs (Hou et al., 2019; Yang et al., 2020). After entering the alveoli, silica dust induces the aggregation and phagocytosis of AMs. Moreover, the intracellular silica dust can also damage the phagosomes and other organelles, such as endosomes, lysosomes, endolysosomes, even DNA, thus inducing cell injury and necrosis or apoptosis (Hou et al., 2019; Yang et al., 2020). After cell death, intracellular silica particles are released together with intracellular enzymes, leading to further cell death and tissue damage (Gao et al., 2020). The above process is caught in a vicious circle. Meanwhile, the secretion of inflammatory mediators activated by AMs can lead to the recruitment of peripheral blood monocytes, which subsequently differentiates into macrophages. Some of these monocytes migrate into the lymph nodes through lymphatic vessels, while others infiltrate the pulmonary interstices (Kawasaki, 2015; Wang et al., 2016; Mu et al., 2020). In addition, silica dust-induced ROS can also result in mitochondrial dysfunction, forcing AMs to undergo mitochondrial apoptosis (Aloe et al., 2022; Nardi et al., 2018).

Inhaled silica dust can affect alveolar epithelial cells by triggering an inflammatory response mediated by AMs. This response leads to degeneration, swelling, and shedding of alveolar epithelial type I (ATI) cells, ultimately resulting in an incomplete alveolar structure (Fang et al., 2018). Alveolar epithelial type II (ATII) cells with the stem cell properties can replenish the lost ATI cells through differentiation and proliferation to restore the alveolar integrity (Wang et al., 2021). If ATII cells are unable to fill the loss timely, the underlying basement membrane of ATI cells will be compromised, exposing the matrix and triggering fibroblast proliferation (Fang et al., 2018).

**FIGURE 2:**
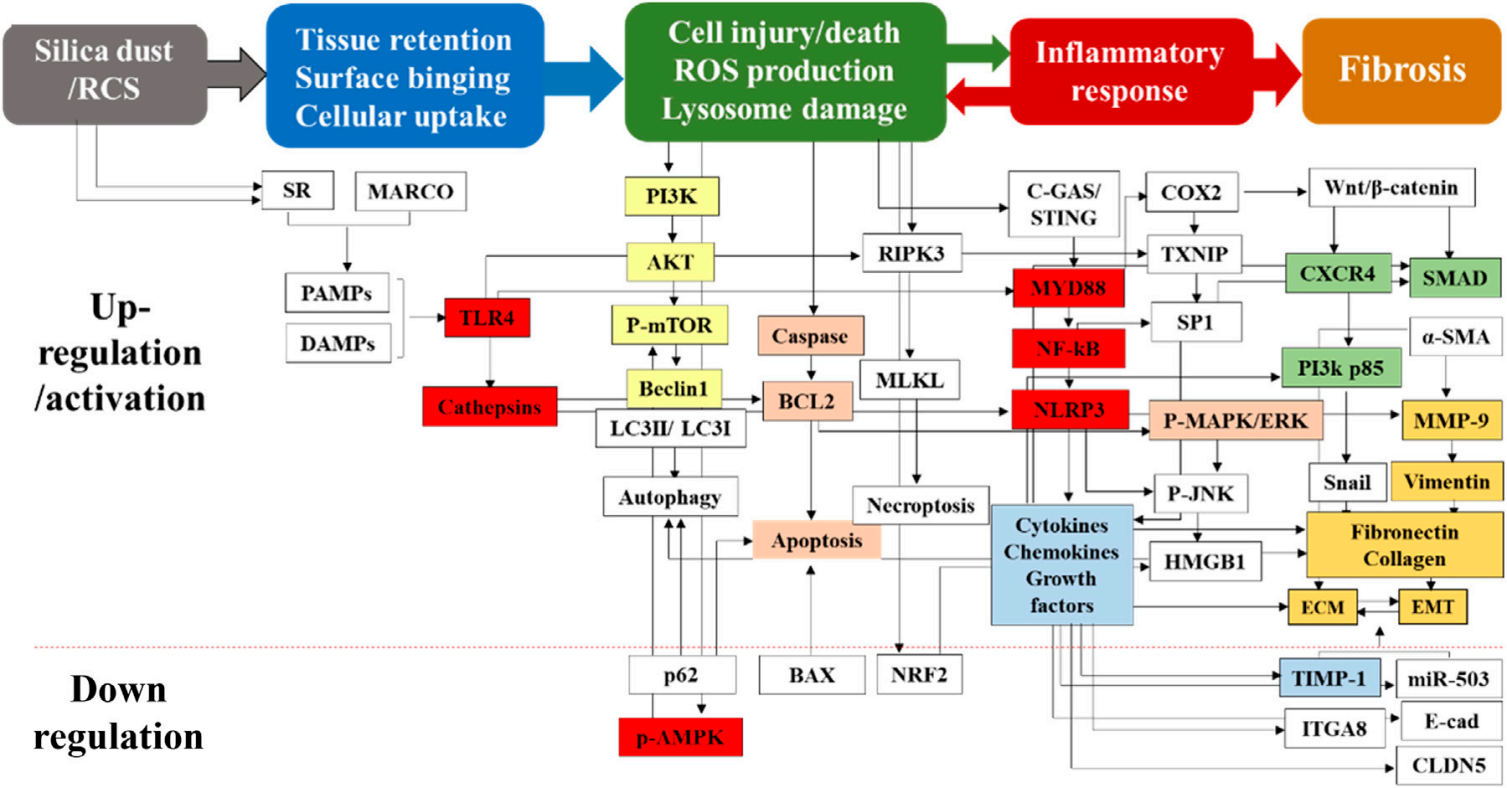
Multiple proteins and genes participate in the pathological process of silicosis. Silica-induced cell and tissue damage is the initial but critical stage for the pathogenesis of silicosis. Moreover, silica dust can activate the apoptosis signaling pathways of AMs and induce cell apoptosis. It can also act on alveolar epithelial cells to cause degeneration, swelling and shedding of alveolar epithelial type I cells, resulting in incomplete alveolar structures. Multiple proteins and genes are involved in above stages. This figure is explained in the part 3 (3. The pathogenesis of silicosis) and part 4.1 (4.1. Drug treatment).

Multiple proteins and genes are involved in the regulation of parenchymal lung injury. Cathepsin participates in fibrosis by degrading the alveolar basement membrane and regulating immune response (Xu et al., 2012). Toll-like receptor 4 (TLR4) is involved in immune response through multiple pathways. Zanoni et al. demonstrated that CD14 was required for TLR4 endocytosis to activate the downstream signaling (Zanoni et al., 2011). The CD14 core focusing defect could inhibit TLR4 endocytosis and impair TLR4 signaling pathways in mouse embryonic fibroblasts (Iijima et al., 2017). This receptor was also found to be critical for the release of pro-inflammatory cytokines and for promoting the deposition of collagens and fibronectins after bleomycin exposure (Razonable et al., 2006; Yang et al., 2009). Therefore, TLR4 plays a key role in the release of inflammatory factors, which provides a basis for the formation of silicotic nodules and pulmonary fibrosis. CLDN5 is an indicator of endothelial tight junctions and permeability, and its downregulation is associated with disruption of endothelial tight junctions in bleomycin-induced pulmonary fibrosis and may be involved in epithelial-mesenchymal transition (EMT) (Zhang et al., 2015). Furthermore, TGF-β also disrupts alveolar epithelial and endothelial tight junctions by downregulating CLDN5 expression (Ohta et al., 2012). ROS is mainly produced by NADPH oxidase (NOX) in AMs. Choi et al. identified that the subunits of NOX complex, such as NOX2 (gp91^phox^), P22^phox^, P47^phox^, P40^phox^, and P67^phox^, were upregulated in silicosis rats. ROS destroyed pulmonary endothelial integrity and increased vascular permeability through activation of P38 MAPK signaling pathway (Choi et al., 2019) (Figure 2, tissue retention and cell injury/death).

### 3.2 Inflammatory response

Silica dust enters the alveoli and interstitium of the lungs, producing toxic effects on the tissues. This exposure activates macrophages, which subsequently interact with alveolar epithelial cells to cause cellular dysfunction even cell death, thereby initiating the inflammatory response. This process represents the primary link in the development of pulmonary fibrosis. The continuous phagocytosis and release of silica dust by macrophages constitute a critical event that contributes to the amplification of inflammation and initiation of pulmonary fibrosis. It is believed that the phagocytosis and consequent lysosomal damage and rupture, ROS, as well as cell injury and death can trigger activation of nucleotide-binding oligomerization domain, leucine-rich repeat and pyrin domain-containing protein 3 (NLRP3) in AMs, which plays an essential role in silica dust-induced inflammatory response (Hamilton et al., 2008; Li Z. et al., 2021). NLRP3, which is composed of a C-terminal leucine-rich repeat domain, a central NACHT domain and an N-terminal pyrindomain (Shen et al., 2021), plays a fundamental role in various inflammation-related diseases, including diabetes, atherosclerosis, metabolic syndrome, cardiovascular and neurodegenerative diseases (Adamcakova and Mokra, 2021; Zahid et al., 2019). Activation of NLRP3 primarily involves two steps: priming or initiating step and oligomerization of NLRP3, as well as subsequent assembly of inflammasomes in which NLRP3 inflammasome binds to an adaptor protein (apoptosis-associated speck-like protein with a caspase recruit domain) to activate the caspase-1 (Yang et al., 2022). When caspase-1 is activated, immature, pro-forms of interleukins (IL)-1β, IL-18, and IL-33 are cleaved to mature and active forms, thereby facilitating various inflammatory processes, including fever, T-cell survival extension, B-cell proliferation, mediation of leukocyte transmigration, etc. (Sims and Smith, 2010). These chemokines and cytokines directly or indirectly cause acute and chronic inflammation, and meanwhile the latter can also induce a variety of particle- and fiber-related lung and pleural diseases (Sayan and Mossman, 2016). Another important function of inflammasomes is the induction of caspase-1-dependent pyroptosis, a highly inflammatory type and programmed cell death characterized by apoptotic and necrotic features (Stegmann et al., 2015). However, the role of pyroptosis in the initiation and development of silicosis remains to be fully understood.

For activation of NLRP3 inflammasome, a priming step is required through activation of pathogen-associated molecular patterns, damage-associated molecular patterns (DAMPs) or TLRs via phosphorylation and subsequent activation of NF-κB, which promotes the transcription of NLRP3, proIL-1β, and proIL-18 in the nucleus. Environmental factors such as silica particles may directly act as exogenous DAMPs, or indirectly as endogenous DMAPs through ROS generation to initiate the activation of NLRP3 (Land, 2020). A recent study has shown that the cyclic GMP-AMP synthase (cGAS)-stimulator of interferon genes (STING) mediates the silica dust-induced activation of NLRP3 inflammasome (Benmerzoug et al., 2018). The lysosome proteases and DNA fragments released from injured and dead AMs or lung epithelial cells are sensed by cGAS/STING, consequently modulating the NALP3 inflammasome and subsequent events (Benmerzoug et al., 2018) (Figure 2, Inflammatory response).

Macrophages can undergo remarkable functional plasticity. In the pathological process of silicosis, macrophages are activated as M1-type macrophages, which release a large number of cytokines and chemokines, leading to neutrophil infiltration and activation of innate immune system (Kawasaki, 2015; Zhao et al., 2020). Meanwhile, macrophages act as antigen-presenting cells to mediate cellular immune activation, promote initial T lymphocytes in peripheral lymph nodes into effector T cells, and migrate to the inflammatory site to secrete Thl type of cytokines such as IL-2, IFN-γ and tumor necrosis factor α (TNF-α), thereby aggravating inflammatory damage (Hou et al., 2019). M1 pro-inflammatory cells contribute to infection clearance, and M2 anti-inflammatory cells have a reparative phenotype and contribute to the resolution phase of response to injury (Tang et al., 2019). M1-type macrophages gradually transform into the M2-type, releasing cytokines such as inhibitors of metalloproteinases, TGF-β and platelet-derived growth factors (PDGF) that play an anti-inflammatory and fibrogenic roles (Kawasaki, 2015). Meanwhile, effector T lymphocytes gradually transform to secrete Th2 cytokines like IL-4 and IL-10, which mainly regulate the fibrotic response.

Notably, in the early stage of silicosis, appropriate apoptosis of AMs can remove the damaged cells, reshape the lung tissue structure, and inhibit inflammation, but this effect disappears in the later stage due to excessive apoptosis (Hou et al., 2019; Yang et al., 2020). Therefore, AMs have the dual effects of defense and inducing fibrotic lung injury. Intracellular silica dust can trigger the release of inflammatory factors via the innate immune system, including IL-1β, TNF-α and transforming growth factor β (TGF-β) (de Lima Gondim et al., 2019). Furthermore, multiple pathways are also induced, such as TGF-β/Smad signaling pathway, NF-κB signaling pathway, etc. (Pang et al., 2021).

Lung epithelial cells play a key role in triggering inflammatory response and promoting remodeling of the lung tissue (Huang et al., 2022). Silica particles can destroy alveolar structures by stimulating alveolar epithelial cells, leading to the release of pro-inflammatory cytokines and recruitment of various inflammatory cells (Mercer et al., 2009). The activation of TLR in epithelial cells may be the trigger for epithelial-induced recruitment of immune cells to the lung (Cho et al., 2022). Importantly, the chemokine response in the lung epithelium can be reinforced by macrophage-derived inflammatory mediators in a synergistic way (Barrett et al., 1998).

### 3.3 Fibrotic changes in the lung

The key pathological change of silicosis is the progressive and irreversible pulmonary fibrosis. During the repetitive inflammatory response, AMs, interstitial and recruited microphages can secrete high levels of cytokines, chemokines and growth factors. Some of the inflammatory mediators can also be fibrogenic factors causing the dysfunction and destruction of epithelium and consequent fibrosis (Ma X. et al., 2020). One of the hallmark changes is EMT, a process in which epithelial cells gradually transform into mesenchymal-like cells and lose the epithelial functions and characteristics (Zhu et al., 2022). Normally, EMT is an important and irreversible process for tissue organization during embryonic development, its dysregulation is associated with a variety of diseases including cancer and fibrosis (Scott et al., 2019). ATII cells play an important role in lung injury through the synthesis and secretion of pulmonary surfactants and conversion into ATI cells as an alveolar repair (Olajuyin et al., 2019). However, due to the cell death and inflammation by silica dust, the differentiation of ATII cells into ATI cells is inhibited, and ATII cells can be transformed into mesenchymal cells or have mesenchymal characteristics, producing extra ECM (Fang et al., 2018; Wang et al., 2021). Normally, ECM provides a supportive environment for cell functions and communications, thus influencing the adhesin, migration and proliferation (Peng et al., 2022).

However, the ECM in the fibrotic lung tissue has abnormal biochemical and biomechanical characteristics, leading to increased hardness of the lung tissue and decreased elasticity, thereby reducing lung function (Aydemir et al., 2022). EMT is influenced and regulated by the surrounding ECM (Scott et al., 2019). Therefore, silica particle-induced aberrant EMT and ECM and their interactions cooperatively induce tissue remodeling. A recent study has shown that EMT, a direct contributor to the fibroblasts transforming into myofibroblasts (Li et al., 2016), is considered the main effector cells in silicosis (Li S. et al., 2019). Myofibroblasts are derived from lung intrinsic fibroblasts, bone marrow fibroblasts, or other mesenchymal cells under the action of cell growth factors (Li et al., 2018). TGF-β1 plays an important role in the trans-differentiation of all the above cell types (Piera-Velazquez et al., 2016; Chen et al., 2011). Besides, myofibroblasts are contractile and can synthesize smooth muscle actins including α-SMA, and their contraction can lead to the structural deformation of lung parenchyma (Leask, 2010; Wang R. et al., 2022). In the normal repair process, the ECM protein will be degraded after eliminating the damage and inflammation, thereby restoring the normal structure of the tissue. However, in the pathological process of silicosis, myofibroblasts can secrete a large amount of ECM and deposit at the injured site (Liu et al., 2017) (Figure 2, Fibrosis).

**FIGURE 3:**
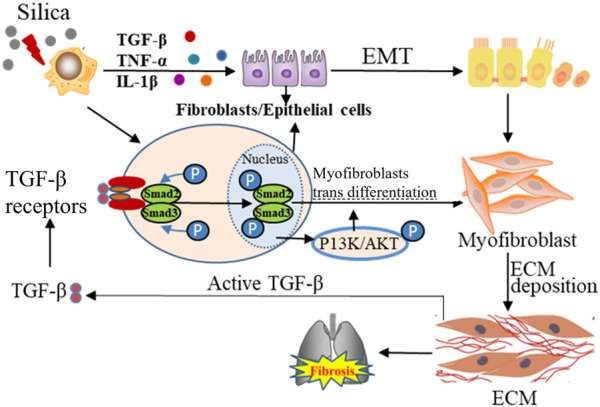
The dynamic interplay between the lung ECM and resident cells, with TGF-β1 as the center. The active TGF-β1 protein in red is attached to the ECM. The activation of this receptor results in the phosphorylation of SMAD2/3, causing its translocation to the nucleus, consequently resulting in the differentiation of the cells into myofibroblasts. The cyclical relationship between TGF-β1 activation via myofibroblasts and the resulting myofibroblast differentiation can be visualized through the arrows.

EMT is regulated by multiple growth factors, ILs, and inflammatory mediators, among which TGF-β has been the most studied growth factor as a central mediator of tissue fibrosis and an inducer of EMT (Pardali et al., 2017). TGF-β receptors can activate Smad-dependent and Smad-independent pathways. In the Smad-dependent pathway, type II TGF-β receptors are activated by ligands and phosphorylate type I TGF-β receptors to form the SMAD complex, which then enters the nucleus and subsequently activates or inhibits the important transcription factors for EMT (Nguyen et al., 2018). In Smad-independent pathway, PI3K/AKT pathway is activated, and PI3K-activated mTORC2 has been identified to be one of the drivers for the phenotypic transformation of EMT. It has been found that inhibition of AKT leads to downregulation of intracellular SNAIL activity and inhibition of EMT (King et al., 2015). In addition, TGF-β-induced activation of the Ras-Erk MAPK pathway, p38 MAPK and JNK signaling, Rho GTPase signaling, as well as PI3K/AKT pathways is all shown to contribute to EMT (Pardali et al., 2017; Gonzalez and Medici, 2014). Although EMT is necessary for proper re-epithelialization and ECM deposition, an uncontrolled, continuous transition from epithelial cells to myofibroblasts can result in fibrosis (Figure 3).

TGF-β1 is also reported to mediate the activation and differentiation of myofibroblasts (Chen et al., 2022). Aberrant ECM mechanical force can induce the release of TGF-β1, further promoting the fibrosis (Upagupta et al., 2018). Recent studies have indicated that the TGF-β signaling pathway can promote the occurrence and development of pulmonary fibrosis by regulating the expression of non-coding RNA molecules and epigenetic modification, which can be the potential target for pulmonary fibrosis (Zhang et al., 2021a; Bartczak et al., 2020).

**FIGURE 4:**
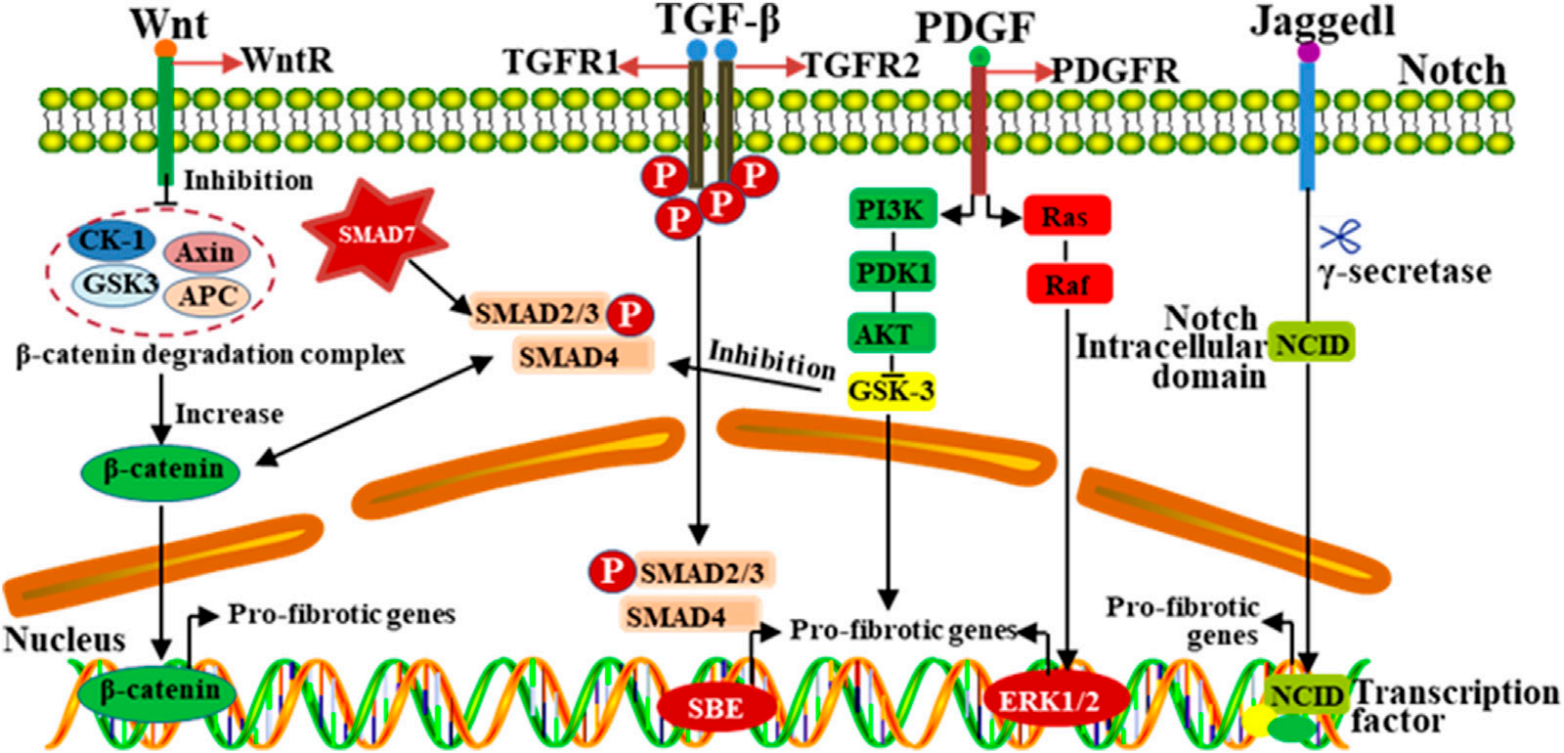
The progression of silicosis is regulated by multiple signaling pathways. Different signaling pathways play complex roles in the regulation of fibrosis, and interact with other signaling pathways in a complex way. Signaling pathways, such as TGF-β, Wnt, MAPK and Notch, have the mutual crosstalk, which jointly participate in regulating the development of pulmonary fibrosis induced by silica dust.

The occurrence and development of silicosis involves multiple signal pathways, including TGF-β, Wnt/β-catenin, Notch and AMP-activated protein kinase (AMPK) pathways, etc. (Feng et al., 2019). Wnt/β-catenin signaling pathway can inhibit the lung fibroblast apoptosis, promote cell proliferation and differentiation (Ma J. et al., 2020). It can also promote ECM deposition by inhibiting glycogen synthase kinase 3-mediated phosphorylation and β-catenin degradation (Gonzalez and Medici, 2014). In addition, the Wnt/β-catenin signaling pathway can cooperate with TGF-β1 to induce the deposition of ECM, thus promoting the production of extracellular matrix metalloproteinase inducers and variations in fibroblast activity. Activation of the Notch pathway could promote the expression of collagens and α-SMA in alveolar epithelial cells (Martins et al., 2020). During the process of EMT, Notch/CSL activation could stimulate the expression of α-SMA in vascular smooth muscle cells. In addition to directly regulating the differentiation of myofibroblasts, Notch could also interact with other signaling (Wnt, TGF-β, PDGF, etc.) pathways to regulate the pulmonary fibrosis (Figure 4) (Feng et al., 2019).

## 4 Treatment of silicosis

### 4.1 Drug treatment

There are no effective drugs for silicosis at present. The drugs for fibrosis including idiopathic pulmonary fibrosis (IPF) are usually used to treat silicosis, such as PVNO, nintedanib, and pirfenidone (Zhao et al., 1983). The *in vivo* and *in vitro* research showed that PVNO could improve the lung clearance after exposure to silica dust and prevent dust from invading the lung interstitium (Ernst et al., 2002). Meanwhile, PVNO also could eliminate the free radicals induced by SiO_2_ and protect macrophages, ultimately reducing the silicosis nodules and delaying the development of silicosis. It has been found that PVNO has a protective effect on silica-induced pulmonary fibrosis, and shows good effects in animal models but no obvious effects in silicosis patients (Idec-Sadkowska et al., 2006).

Nintedanib, an orally administered multi-target agent, was approved for IPF in the United States in 2014 and in Europe in 2015 (Varone et al., 2018). Its main molecular targets are the fibroblast growth factor receptors, PDGF receptors and vascular endothelial growth factor receptors (Varone et al., 2018). Due to potentially similar pathological characteristics between IPF and the same tyrosine kinase receptors in progressive fibrosis (Allen and Spiteri, 2002; Coward et al., 2010), nintedanib had been tested in murine models of bleomycin- and silica-induced pulmonary fibrosis, where it was demonstrated to reduce or prevent the fibrotic process (Chaudhary et al., 2007; Wollin et al., 2015). Additionally, nintedanib can also inhibit PDGF receptor activation, fibroblast proliferation, and fibroblast-to-myofibroblast transformation (Wollin et al., 2014). These results indicate that nintedanib may impact the progression of fibrotic lung diseases, such as silica-induced pulmonary fibrosis.

Pirfenidone is an oral anti-fibrotic agent, although it was initially developed as an anti-inflammatory compound due to its capability of diminishing accumulation of inflammatory cells and cytokines (Lancaster et al., 2017). So far, the precise mechanisms underlying the anti-fibrotic action of pirfenidone in the lung are not fully understood. Some research demonstrate that pirfenidone may attenuate the fibroblast proliferation, myofibroblast differentiation, collagen synthesis, fibronectin production and deposition of ECM through modulation of fibrogenic growth factors (Ma Z. et al., 2018; Qin et al., 2018; Molina-Molina et al., 2018; Pourgholamhossein et al., 2018). Additionally, pirfenidone also could regulate and reduce oxidative stress markers (ROS, H_2_O_2_, etc.) in the lung, which might be associated with its anti-fibrotic effect (Pourgholamhossein et al., 2018; Gaggini et al., 2011). A retrospective study including 186 subjects who were continuously treated with pirfenidone or nintedanib for pulmonary fibrosis (of any cause) showed similar drug tolerability and adverse event profiles to the corresponding clinical trials, despite the presence of more severe respiratory impairment (Galli et al., 2017). Fortunately, both pirfenidone and nintedanib are promising in the treatment of chronic lung inflammatory diseases, pulmonary fibrosis and other similar diseases. However, future prospective studies are still needed to elucidate the efficacy of the drugs with expanded prescription.

Drug repurposing or repositioning for different diseases is an efficient way for drug discovery because of low cost in the drug development (Pushpakom et al., 2019). Metformin, a widely used biguanide medication for type 2 diabetes, has been shown to inhibit cardiac fibrosis induced by pressure overload *in vivo*. It may reduce collagen synthesis in cardiac fibrosis by inhibiting the TGF-β/Smad3 signaling pathway (Xiao et al., 2010). Moreover, metformin has antifibrotic properties (Teague et al., 2022). It can effectively reverse bleomycin-induced pulmonary fibrosis, suggesting its role in IPF (Gamad et al., 2018). In our previous study, metformin has been identified to have anti-silicosis effects whether in rats or *in vitro* cultured human cells (Li S. X. et al., 2021). Metformin could regulate autophagy by activating AMPK and inhibiting mTOR pathways, providing the evidence for metformin as the potential therapeutic drug for silicosis.

In recent years, a number of traditional Chinese medicine compounds have been applied to treat pulmonary fibrosis, including silica-induced pulmonary fibrosis (Li and Kan, 2017; Occupational Lung Disease Group of Labor Hygiene, 2024). These compounds/molecules exert their effects through targeting different pathways as summarized in Figure 2. Liu et al. found that Number 2 Feibi Recipe (N2FBR) with antioxidant effects could promote autophagy through the GSK-3β/mTOR signaling pathway, thereby exerting a protective effect on pulmonary fibrosis (Liu et al., 2022). Xiaochaihu decoction showed anti-fibrotic functions by reducing the collagen content and fibrogenic score, as well as down-regulating TGF-β1, PDGF and TIMP-1 mRNA levels (Figure 2, Orange) (Chen et al., 2004; Chen et al., 2005). Fuzheng Huayu Formula could block the PI3K pathway related to the progression of liver fibrosis, and downregulate the expression of TGF-β1 and Smads (Li, 2020) (Figure 2, Green). Curcumin could suppress EMT and inflammatory response via inhibition of TLR4/NF-κB and PI3K/AKT pathways (Figure 2, Yellow) (Wang Z. et al., 2020)]. Anluo Huaxian Pills could reduce the expression of collagens I and III, TIMP-1, and TGF-β1 in mice with liver fibrosis (Xie et al., 2018) (Figure 2, Blue). Moreover, treatment with salidroside could also significantly decrease the release of inflammatory cytokines (IL-1β, IL-6, TNF-α) and inhibit TLR4/NF-κB and MAPK signaling pathways (Figure 2, red) (Li R. et al., 2019).

Tetrandrine, a bis-benzyl iso-quinoline alkaloid extracted from the plant called *Stephania tetrandra S. Moore* (Su W. et al., 2020), has been approved for the long-term treatment of silicosis (Liu et al., 2016). Tetrandrine can effectively inhibit the transcription of collagen genes, weaken the function of collagens before cell secretion, reduce the synthesis and proliferation of collagen fibroblasts, and degrade lung collagen fibers, thereby delaying the progression of silica-induced pulmonary fibrosis (Su W. et al., 2020; Xie et al., 2002; Droitcourt et al., 2018). Meanwhile, the compounds from Chinese herbs, such as ginsenoside Rg1, Baicalin, and more, can also reduce the degree of pulmonary fibrosis and improve lung function (Yang et al., 2016; Yu et al., 2016; Liu et al., 2015). Notably, the anti-silicotic effect of emodin was also observed in our *in vivo* and *in vitro* studies, indicating that emodin could alleviate silica dust-induced pulmonary fibrosis through regulation of the inflammatory response and fibrotic process at multiple levels (Pang et al., 2021).

Traditional Chinese medicine has been demonstrated effective in various diseases, with fewer adverse effects, which contributes to alleviating the clinical symptoms of patients and enhancing their QOL (Kong et al., 2022; Zhang et al., 2021b; Zhang et al., 2022). In addition, Chinese herbal formulae have been widely prescribed as an adjunct to western medicine to treat the disease (Li and Kan, 2017), which may be the future direction of drug therapy.

### 4.2 Surgical treatment

#### 4.2.1 Bronchoalveolar lavage

Bronchoalveolar lavage is mainly used to reduce the number of dust particles in the lungs and improve the ventilation function of lung tissues (Cottin et al., 2004). Alveolar lavage with 37°C saline could not only remove the silica particles depositing in the alveoli and the lung interstitium effectively, but also remove the macrophages in the alveoli and slow the progression of silicosis. After bronchoalveolar lavage, cough and discharge of foreign bodies are enhanced, thereby improving tracheal obstruction and ventilation function. Meanwhile, the bronchoscope can be used locally to improve the availability of drugs, reduce local inflammation, and improve the lung function of patients. According to the amount of lavage fluid, bronchoalveolar lavage can be divided into the WLL and small volume lung lavage, among which small volume lung lavage is mainly suitable for the patients with advanced silicosis who are unable to receive WLL. Notably, bronchoalveolar lavage can cause a high risk of trauma. More studies are required to confirm its long-term efficacy.

#### 4.2.2 Lung transplantation

Lung transplantation has become an accepted treatment option for patients with various lung diseases irresponsive to conservative treatments (George et al., 2019). It can improve the QOL of patients, but cannot extend the survival. Although lung transplantation is the most effective method for end-stage fibrosis, its application is limited due to a shortage of liver donors, high incidence rates of surgical complications, graft-versus-host diseases and high medical costs (Hartert et al., 2014). Therefore, similar to bronchoalveolar lavage, lung transplantation should not be used as a routine treatment for silicosis.

### 4.3 Stem cell treatment

Mesenchymal stem cell (MSC)-based cell therapy is regarded as an innovative experimental treatment (Maria et al., 2018). MSCs, a population of multipotent stem cells, are originated from various tissues and organs, including bone marrow, adiposes, cord blood, and placentae. Multipotentiality is one of the properties for these cells that not only differentiate into adipocytes, chondrocytes and osteoblasts, but also vascular smooth muscle cells and lung epithelial cells under particular conditions (Li et al., 2017). At present, the anti-fibrotic effects have been demonstrated in several types of MSCs, such as bone marrow-derived MSCs (BMSCs), umbilical cord mesenchymal stem cells (UC-MSCs), Sox9^+^ embryonic stem cells and adipose-derived MSCs (AD-MSCs).

Based on the capability of differentiating into specific cell types, BMSCs may promote the tissue regeneration (Peng et al., 2015), and have immunomodulatory and anti-fibrotic activities that can be significant in response to injury. In animal models of pulmonary fibrosis, transplanted BMSCs home to sites of injury (Xu et al., 2015), which can ameliorate the histological alterations (Ni et al., 2015), inhibit production of proinflammatory mediators (Xue et al., 2013), and decrease collagen deposition (Lan et al., 2015). In addition, BMSCs also attenuate lung injury and pulmonary fibrosis by secreting various factors with anti-apoptotic, anti-inflammatory, and anti-fibrotic functions. Due to easy harvest, isolation and purification, BMSCs have been considered to be a promising and novel treatment (Maria et al., 2018). Our previous study showed that BMSC transplantation could relieve silica-induced pulmonary fibrosis in rats through attenuation of the Wnt/β-catenin signaling pathway (Zhang et al., 2018).

Unlike BMSCs, UC-MSCs are characterized by a painless collection process and a faster self-renewal (Tsai et al., 2021). Inhibition of inflammation is one of the mechanisms for UC-MSCs to treat silicosis. Sha et al. found that UC-MSCs could effectively reduce SiO_2_-induced inflammatory cell infiltration and inflammation-related cytokine levels in the lung, thereby reducing fibrosis (Sha et al., 2019). UC-MSCs also might alleviate the degree of pulmonary fibrosis in the rat model of silicosis by regulating the secretion of hydroxyproline, TGF-β1 and IL-6 (Xu et al., 2020). In addition, UC-MSCs could also play a role in the treatment of silicosis by reducing cell apoptosis (Chen et al., 2018b), inhibiting the autophagy of lung macrophages, and enhancing the repair after injury (Tuo et al., 2017).

Embryonic stem cells with an enormous capacity for regeneration can differentiate into a variety of tissues (Wu et al., 2020). Sox9^+^, one of the key genes in early embryonic development, is closely related to cell proliferation and differentiation, and plays a role in balancing and regulating the homeostatic maintenance and directional differentiation of stem cells (Gonen and Lovell-Badge, 2019; Hersmus et al., 2008; Jo et al., 2014). The normal expression of Sox9^+^ gene determines the integrity of embryonic lung development (Rockich et al., 2013). Lung stem cells involved in lung regeneration and repair are closely related to the process of lung development (Chen et al., 2021). Ma et al. found that after transplantation of autologous Sox9^+^ airway basal cells for 3–12 months, the lung tissue was repaired, and lung function was enhanced (Ma Q. et al., 2018). In addition, Nichane et al. showed that in the mouse with bleomycin-induced lung injury, endotracheal transplantation of mouse Sox9^+^ embryonic lung progenitor cells could be integrated into the injured lung tissue and mainly differentiated into alveolar epithelial cells (e.g., ATI and ATII cells), endothelial cells and mesenchymal cells (Nichane et al., 2017). These studies indicated that Sox9^+^ embryonic lung stem cells could be integrated into the injured lung tissue, with the ability to colonize and differentiate into lung epithelial cells. Meanwhile, in clinical trials, autologous Sox9^+^ stem cell transplantation is also reported to have the ability to treat lung injury, bringing hope for the treatment of pulmonary fibrosis.

Autologous AD-MSCs with multi-directional differentiation potential can be obtained from mature adipose tissues (Chen et al., 2018b), and differentiate into adipocytes, osteocytes, chondrocytes, muscle cells and nerve precursor cells (Taghi et al., 2012; Nakao et al., 2010). They are abundant in adipose tissues, easy to obtain, relatively less painful and highly feasible (Chen et al., 2018b), which ensure that AD-MSCs have a wider range of applications in stem cell treatment. Studies have shown that Ad-MSCs can effectively repair and regenerate lung tissues (Zhang et al., 2014; Wang et al., 2013) and improve IPF (Jiang et al., 2015). Rubio et al. demonstrated that AD-MSCs could mitigate bleomycin-induced tissue damage and prevent terminal organ fibrosis, manifesting as attenuation of lung and skin fibrosis, as well as acceleration of wound healing (Rubio et al., 2018). In addition, AD-MSC transplantation may also interfere with the formation of silicosis by regulating the processes of inflammation and apoptosis (Chen et al., 2018b).

For silicosis, stem cells can be promising candidates. However, before the cells can be transferred to clinical research from basic research, several issues remain to be resolved: 1) Establishing regulatory guidelines and efficient, safe manufacturing procedures; 2) Establishing a system for genetic testing and long-term monitoring of donors; 3) Implementation of clinical trials to determine the best and standard dose, time, approach, frequency, and other technical issues of stem cell transplantation.

## 5 Conclusion

Although the etiology of silicosis is clear, the exact pathogenesis is not fully understood. The pathological process of silicosis is complex, involving the interaction of multiple cells and molecules, as well as abnormal regulation of multiple signal pathways and cytokines. Several pathways or mediators playing a key role in initiation and development of silicosis have been proposed to be potential therapeutic targets, and meanwhile surgical treatments and compounds have been used for silicosis. However, the actual clinic effect of these methods on silicosis and possible applications have a long way to go. Early diagnosis and timely monitoring are important strategies in the treatment of silicosis. Currently, the examinations used to diagnose silicosis include chest X-rays, computed tomography scans, and lung function testing. However, such tests only recognize the pathological changes at an organ level. When there have been substantial fibrotic changes, no reliable methods are applied for the diagnosis of silicosis, especially for accelerated silicosis. Although several biomarkers of silicosis have been proposed, their reliability and sensitivity remain to be confirmed and none of them is reported in early diagnosis. Therefore, more work should be focused on the identification of reliable and feasible biomarkers or the methods for earlier diagnosis or health surveillance. To this end, *in vitro* research in combination with epidemiological data is required with feasible biological samples such as blood, saliva/sputum, and/or urine samples.
